# Astaxanthin attenuates CTX-induced premature ovarian failure by alleviating ovarian apoptosis and autophagy in vivo

**DOI:** 10.1186/s13048-026-02175-x

**Published:** 2026-07-03

**Authors:** Ling Nie, Zihang Wang, Xinyuan Huang, Yating Yu, Jia Li, Han Zeng, Dingfei Xu

**Affiliations:** 1 Department of Reproductive Medicine, The First Affiliated Hospital, Jiangxi Medical College, Nanchang University, Nanchang, 330006 People’s Republic of China; 2 School of Basic Medical Science, Jiangxi Medical College, Nanchang University, Nanchang, 330031 People’s Republic of China; 3 The Second School of Clinical Medicine, Jiangxi Medical College, Nanchang University, Nanchang, 330031 People’s Republic of China; 4 Jiangxi Medical College, HuanKui Academy, Nanchang University, Nanchang, 330031 People’s Republic of China

**Keywords:** Premature ovarian failure, Autophagy, CYP19A1, Astaxanthin, Ovarian function

## Abstract

**Background:**

Cyclophosphamide (CTX) is a commonly used chemotherapeutic agent for breast cancer that frequently causes premature ovarian failure (POF), a clinical syndrome characterized by menstrual irregularities in women under the age of 40 years, accompanied by elevated serum follicle-stimulating hormone (FSH) and decreased estrogen levels. Astaxanthin (AS), a natural antioxidant, has been shown to exert various biological effects, including anti-aging and anti-inflammatory effects. However, further investigation into its anti-ovarian aging mechanism is warranted.

**Methods:**

Two-month-old female mice and CTX-induced POF model mice were used. Hematoxylin and eosin staining, immunohistochemical staining, TUNEL assays, Western blotting, and qPCR analyses were employed to evaluate ovarian function and related phenotypes after astaxanthin treatment. Subsequently, network pharmacology analysis was used to elucidated potential targets.

**Results:**

Astaxanthin intervention significantly ameliorated estrous cycle disorder in POF mice and restored serum levels of anti-Müllerian hormone (AMH) and estradiol (E_2_). Meanwhile, astaxanthin markedly enhanced the ovarian reserve and suppressed CTX-induced apoptosis and autophagy. Mechanistically, astaxanthin was found to modulate the CYP19A1 expression, thereby enhancing ovarian function.

**Conclusion:**

This study elucidates the mechanism by which astaxanthin improves ovarian function through the CYP19A1, providing potential molecular targets and therapeutic strategies for the clinical treatment of POF.

**Supplementary Information:**

The online version contains supplementary material available at https://doi.org/10.1186/s13048-026-02175-x.

## Introduction

Premature ovarian failure (POF) is a clinical syndrome in which woman develop menstrual dysfunction associated with elevated follicle-stimulating hormone (FSH) and decreased estrogen levels before the age of 40 [1–3]. Chemotherapy is a major iatrogenic causes of POF in women of childbearing age [4, 5]. Cyclophosphamide (CTX) is a broad-spectrum anti-tumor drug, but it also has obvious toxicity to the reproductive system, which can cause menstrual disorders, azoospermia or reduced sperm count, and has mutagenic and teratogenic effects [6, 7]. CTX-induced POF models are commonly used to simulate the pathological process of ovarian damage caused by chemotherapy, and are highly clinical relevant. Therefore, there is an urgent need to elucidate the mechanism and etiology of CTX-induced POF, which would facilitate the development of effective preventive and therapeutic strategies to preserve ovarian function.

Astaxanthin (AS), a natural carotenoid widely distributed in microorganisms and marine organisms, is a potent antioxidant with diverse biological activities, including anti-aging and anti-inflammatory properties [8, 9]. A substantial corpus of research has demonstrated astaxanthin enhances the follicular development in vitro, alleviates oxidative stress in preantral follicles, and promotes mitochondrial function during in vitro culture [10, 11]. Moreover, astaxanthin has been reported to regulate cellular autophagy, enhance the resilience of cells to oxidative stress, and retard the progress of aging [12–14], AST also can enhance mitochondrial function through the activation of the nuclear factor (erythroid-derived 2)-like 2(Nrf2)/ Heme Oxygenase-1(HO-1) pathway, thereby promoting autophagy [15].

Autophagy is a process mediated by lysosomes for the clearance of protein aggregates and damaged organelles, and it is a dynamic and continuous process [16]. Many studies have indicated that autophagy exerts dual regulatory effects in the pathogenesis of numerous diseases [17, 18]. The physiological level of autophagy is usually relatively low, which promotes cell survival. However, either insufficient or excessive autophagy can lead to cell death [19, 20]. Studies have found that autophagy is related to the preservation of the primordial follicles that form the ovarian reserve. Increasing evidence suggests that autophagy induces apoptosis by triggering the accumulation of autophagosomes in granulosa cells and luteal cells [21].

CYP19A1 is predominantly expressed in ovarian granulosa cells, where it mediates local estrogen synthesis [22–24]. Estrogen, in turn, exerts pleiotropic effects on GC survival and function. Recent studies have uncovered a bidirectional crosstalk between estrogen signaling and autophagy. CYP19A1 may serve as a molecular hub that integrates hormonal signaling and autophagic homeostasis in GCs [25]. The aim of this study was to investigate whether AST improves ovarian reserve function in CTX-induced POF and to elucidate its underlying molecular mechanism. This study elucidates the molecular mechanism by which astaxanthin protects ovarian function, providing a potential therapeutic target and intervention strategy for the treatment of POF.

## Materials and methods

### Study approval

KM mice were reared according to the National Institutes of Health Guide for the Management and Use of Laboratory Animals, and the Laboratory Animal Ethics Committee of Nanchang University approved the experiments. Six-week-old female KM mice (*n* = 24) were purchased from the Jiangxi University of Traditional Chinese Medicine. All mice were housed under specific pathogen-free (SPF) conditions at a temperature of 20 ± 2 °C, humidity of 50–70%, and a 12-h light-dark cycle. The reporting of this study adheres to the SQUIRE 2.0 guidelines. The completed checklist is available as supplementary material.

### Animal model

KM mice, the subjects of this experimental study, were housed in the Laboratory Animal Centre of Nanchang University under specific pathogen-free conditions and fed cobalt-60 irradiated and SPF-grade diets. For the induction of the mouse POF model, 6-week-old female KM mice were treated as described in the previous study: 70 mg/kg cyclophosphamide (CTX, C0768, Sigma-Aldrich) and 12 mg/kg leucovorin (BU, B2635, Sigma-Aldrich) were injected intraperitoneally once a week for two weeks. The control group was injected with the same amount of saline [26]. Subsequently, the doses were selected based on a pilot experiment (*n* = 5 per group) comparing 100, 200, and 300 mg/kg astaxanthin, with the optimal dose defined as the lowest dose that produced the maximum improvement in ovarian reserve and hormonal function without adverse effects. After weighing the mice and collecting blood via eyeball puncture, the mice were euthanatized with 100 mg/kg sodium pentobarbital and placed in a supine position. The ovaries and other tissues were then dissected, weighed, photographed, and stored at -80 °C for later use.

### Histological analysis of ovarian tissue and follicle count

We used mouse ovaries fixed in 4% paraformaldehyde solution, dehydrated in ethanol and xylene, and embedded in paraffin. Paraffin-embedded ovaries were serially sectioned at 5 μm thickness using a microtome (RM 2255, Leica, Berlin, Germany). Ovarian sections were then deparaffinized in xylene, rehydrated in ethanol, and stained with H&E. Follicles were classified according to different criteria. The primordial follicles were surrounded by a single layer of flat granulosa cells; primary follicles were surrounded by a single layer of cuboidal granulosa cells and fully enlarged nucleated oocytes were visible; Multiple layers of granulosa cells surrounded secondary follicles without sinusoidal cavities; sinusoidal follicles had sinusoidal cavities and were indistinct from the surrounding connective tissue; and atretic follicles were seen with follicle walls collapsed and the layer of granulosa cells loosened or even absent. Follicle counts were performed and statistically analyzed [27].

### Definition of the phase of the estrous cycle

Vaginal swabs were collected at approximately 9 am each day for 14 consecutive days to determine the stage of the estrous cycle in mice by cytological examination under a microscope. The stages of the estrous cycle were determined by cytological examination under the microscope, and the estrous cycle was categorized as proestrus, estrus, and postestrus [22]. The smears were taken on 14 consecutive days. 

### Measurement of hormone levels

Blood samples were taken from the retro-orbital venous plexus of mice, and the serum was separated. Enzyme-linked immunosorbent assays (ELISA) were then used to determine the levels of estradiol (E2, E-EL-0150c, Elabscience), Follicle Stimulating Hormone (FSH, E-EL-M0511, Elabscience), Luteinizing Hormone (LH, E-EL-M3053, Elabscience), and anti-Müllerian hormone (AMH, E-EL-M3015, Elabscience), strictly following the reagent instructions.

### The ovary index

After the mice were executed, the ovarian tissues were removed under aseptic conditions, rinsed well with PBS buffer, drained of surface liquid, and weighed. The ovarian index was calculated according to the following formula: ovarian index (‰) = (ovarian weight/body weight) × 1000 [28].

### Western blot

Radioimmunoprecipitation assay (RIPA) buffer (R0010, Solarbio, Beijing, China) supplemented with a protease inhibitor cocktail (100×, HY-K0010, MedChemExpress) at a final concentration of 1×. Total protein was extracted from ovarian tissue from ovaries. Total proteins (30 µg) were separated using sodium dodecyl sulfate-polyacrylamide gel electrophoresis (using 12% or 10% gels) and transferred to a polyvinylidene difluoride membrane (IPVH 00010, Sigma-Aldrich, St. Louis, MO, USA). The membrane was then incubated with the appropriate primary antibody: anti-BCL2 (1:2000, ab182858, Abcam, Shanghai, China), anti-BAX (1:2000, ab32503, Abcam, Shanghai, China), anti-Cleaved Caspase3 (1:1,000, 25128-1-AP, Proteintech, China), anti-LC3 (1:2, 000, 14600-1-AP, Proteintech, China), anti-BECLIN1 (1:2,000, 11306-1-AP, Proteintech, China), anti-ATG5 (1:2,000, 10181-2-AP, Proteintech, China), anti-P62 (1:2,000, 10181-2-AP, Proteintech, China), anti-SOD2 (1:5,000, 24127-1-AP, Proteintech, China), anti-HO-1 (1:2,000, 10701-1-AP, Proteintech, China), anti-CYP19A1 (1:1,000, 16554-1-AP, Proteintech, China) for 14 h at 4 °C. Next, the membranes were incubated with horseradish peroxidase (HRP)-conjugated goat anti-rabbit IgG (1:5000, SA00001-2, Proteintech, China) or goat anti-mouse IgG (1:5000, SA00001-1, Proteintech, China). The protein bands were visualized by using an enhanced chemiluminescence detection kit (S6009M, Uelandy, China). Appropriate secondary antibodies were incubated at room temperature for 1 h, and bands were visualized with ImageJ software (National Institutes of Health, Bethesda, MD, USA).

### RNA extraction and real-time quantitative PCR (qRT-PCR)

Total RNA was isolated using TRIzol reagent (Yeasen, 19201ES60), and reverse-transcribed into cDNA using the PrimeScript RT Kit (6210A, Takara Bio, Kyoto, Japan), both according to the manufacturer’s instructions. Quantitative real-time PCR (qRT-PCR) was performed using the SYBR Premix Ex Taq Kit (MF013, Mei5bio, Beijing, China) in triplicate. 2 ΔΔCt method was used for relative quantification of target genes.

### Immunohistochemistry of ovarian tissue

Immunohistochemistry (IHC) was carried out on 5 μm sections of paraffin-embedded tissue. After the slides were baked at 60°C for 2 h, dewaxed, and rehydrated, antigen retrieval was performed. Next the sections were incubated with the corresponding primary antibodies: anti-ATG5, and anti-CYP19A1 for 14 h at 4 °C. Sections were incubated with secondary antibody (goat/IgG, HRP-coupled, PV-6001, Beijing, China) for 1 h. DAB (Beijing, China) staining solution were added for staining. Stained sections were visualized and images were captured under a light microscope.

### TUNEL staining 

Apoptosis in ovarian tissues was assessed utilizing the terminal deoxynucleotidyl transferase dUTP nick-end labeling (TUNEL) technique, as outlined in the accompanying instructions of the kit (Catalog #E-CK-A321, Elabscience Biotechnology, Inc., China). 

### Measurement of SOD, ATP and MDA

We procured the CAT, ATP, and SOD assay kits (Cat. No. A007-1-1, A095-2-1 and A001-3-2, respectively) from Nanjing Jiancheng Bioengineering Institute Co., Ltd. (Nanjing, China) and used them according to the manufacturer’s instructions. We measured ATP accumulation and SOD and CAT activity during the various treatment groups. We used a multimode plate reader to read the absorbance and calibrated the results with the protein concentration.

### ROS level

The ovaries were crushed, and PBS was added. Intracellular ROS levels were determined with the ROS detection kit (#R6033, US Everbright). Briefly, 10 µM 2′,7′-Dichlorodihydro-fluorescein diacetate (DCFH-DA) was added to the ovarian supernatant and incubated at 37 ◦C for 30 min; The suspension was washed one to three times with serum-free medium and then placed into an enzyme-labeled analyzer at a wavelength of 480 nm [29].

### Collection of astaxanthin and POI targets

Submit the standard English name of astaxanthin AST to the PubChem database (https://pubchem.ncbi.nlm.nih.gov/) to obtain its standardized SMILES structural formula. Use the Swiss Target Prediction platform (http://www.swisstargetprediction.ch/), input the SMILES number of AST, and predict its potential target sites in humans (Homo sapiens). In the GeneCards database (https://www.genecards.org/), a search was conducted using the keyword “premature ovarian failure” and the species was limited to “Homo sapiens”.

### Network creation and functional enrichment evaluation

Based on the above Astaxanthin-regulated POF targets, the component-target network was constructed using Cystoscope. Furthermore, the protein-protein interaction (PPI) network of Astaxanthin-regulated POF targets was generated by the STRING database and subsequently displayed using Cystoscope. The Metas cape database were used to conduct functional enrichment analysis, including gene ontology (GO) analysis and Kyoto Encyclopedia of Genes and Genomes (KEGG) pathway. The Venn diagrams, KEGG, and GO visualizations were performed by Bioinformatics.

### Statistical methods

Each experiment was performed with at least three independent biological replicates. All data are expressed as mean ± standard deviation (mean ± SD), and experimental data were analyzed by one-way analysis of variance (ANOVA) followed by Student t-test using GraphPad Prism 8.0.2 (California, USA). Statistically significant differences were considered at *p* < 0.05 (*p < 0.05 was considered significant; **p < 0.01 and ***p < 0.001 were considered highly significant).

## Results

### Astaxanthin effectively improves the reproductive capacity of female mice induced by CTX

**Fig. 1:**
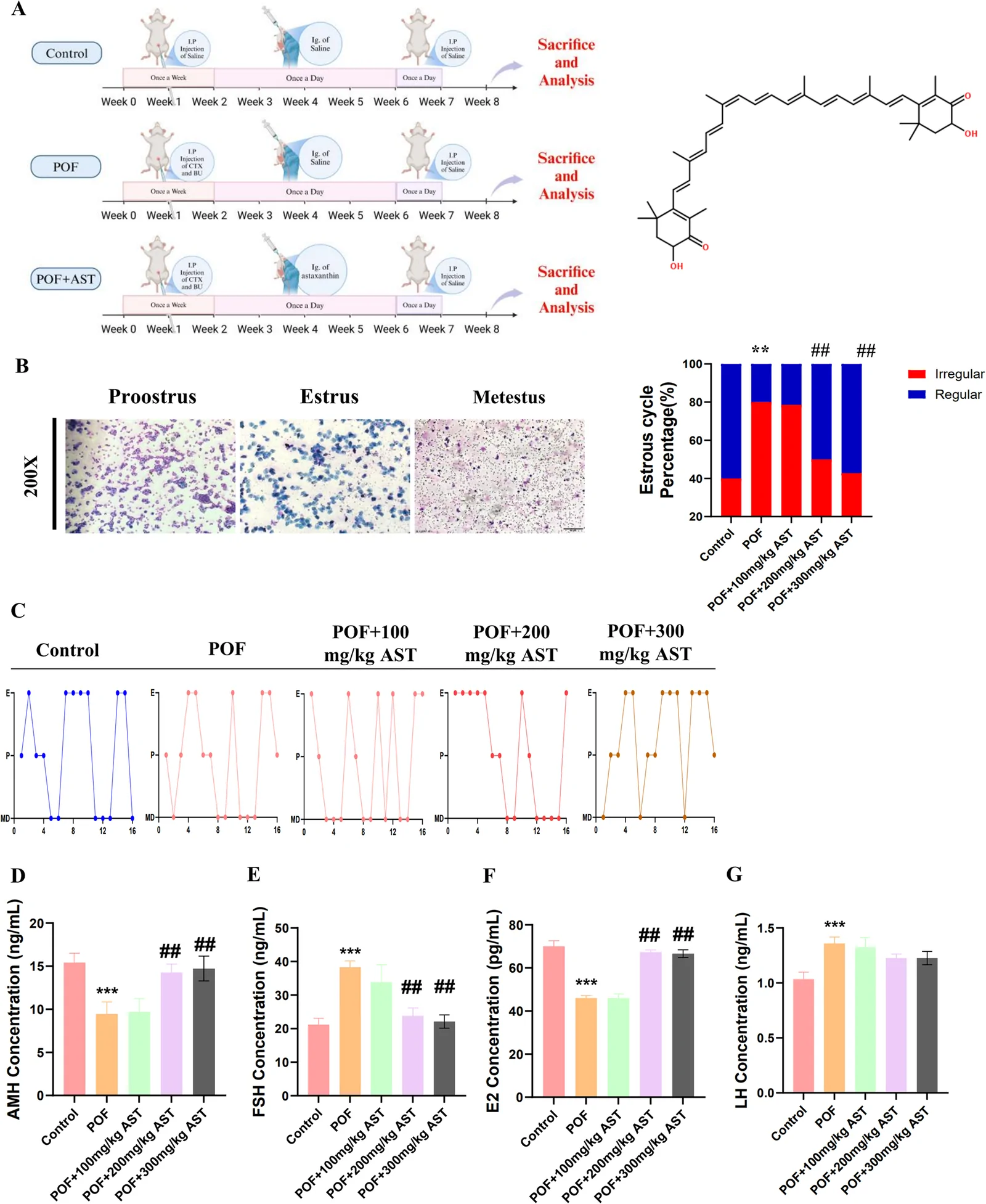
Screening for the optimal drug concentration of astaxanthin to improve reproductive endocrine disorders in mice. **A** Construction of animal models, and the chemical structure formula of astaxanthin. **B** Control group, POF group, and POF + AST group representative estrous cycle plots (*n* = 5), magnification, 200×. **C** Proportion of regular and irregular estrous cycle in Control, POF, and POF + AST groups (*n* = 5). **D**-**G** AMH, FSH, LH and E_2_ levels in peripheral blood of mice in Control, POF, and POF + AST groups (*n* = 5). **p < 0.01, ***p < 0.001compared with the control group, ##*p* < 0.01 compared with the POF group, one-way ANOVA

Astaxanthin exhibits a broad spectrum of biological activities, including potent antioxidant, anti-aging, antitumor, anti-inflammatory, and anti-fatigue effects [8, 9]. However, the optimal dosage required to enhance ovarian reserve function remains to be determined. KM mice are widely recognized as a reliable animal model for reproductive toxicity and CTX-induced POF studies [30, 31]. Therefore, we administered astaxanthin via gavage to KM mice and a murine model of POF mice at doses of 100, 200, and 300 mg/kg/day (Fig. 1A). POF mice exhibited irregular changes in the estrous cycle, while astaxanthin treatment significantly ameliorated estrous irregularity during 200 and 300 mg/kg treatment group compared with the POF group (Fig.1B, C). In addition, serum anti-Müllerian hormone (AMH) levels—reflecting ovarian follicular reserve—were significantly elevated in the 200 and 300 mg/kg astaxanthin groups relative to the POF control (Fig. 1D). By detecting the follicle-stimulating hormone (FSH) levels of different groups, serum FSH levels were significantly increased in the POF group compared with the control group. Astaxanthin treatment at 200 and 300 mg/kg significantly reduced FSH levels, whereas the 100 mg/kg dose had no significant effect (Fig. 1E). Similarly, serum estradiol (E2) levels were significantly decreased in the POF group compared with the control group. Treatment with 200 and 300 mg/kg astaxanthin partially restored E2 levels (Fig. 1F; P < 0.01), whereas the 100 mg/kg dose showed no significant effect. Serum luteinizing hormone (LH) levels were elevated in the POF group relative to controls, and although astaxanthin treatment at 200 and 300 mg/kg induced a modest reduction in LH, this change did not reach statistical significance (Fig. 1G).

###  Astaxanthin effectively ameliorates ovarian reserve dysfunction in a murine model of POF

**Fig. 2:**
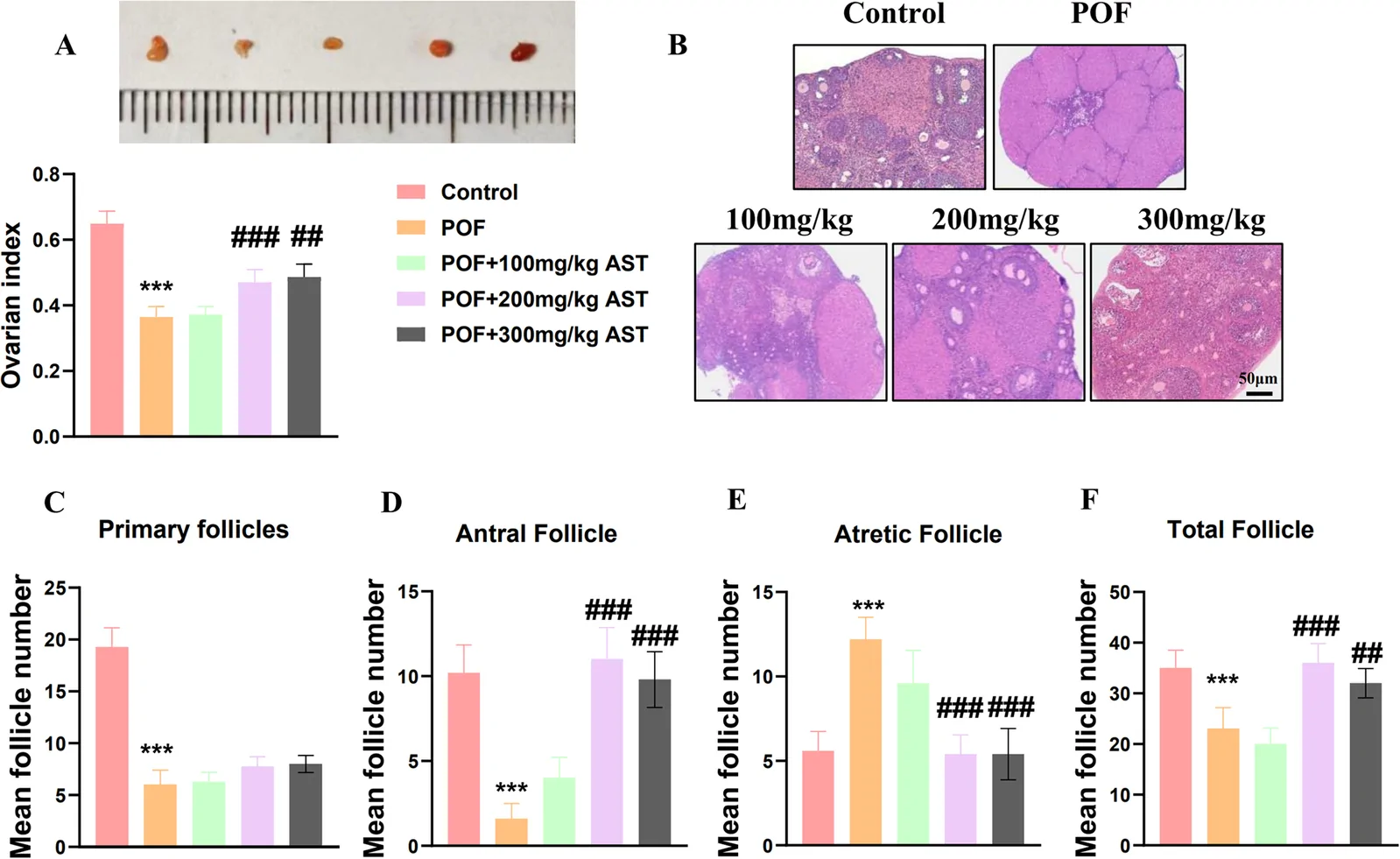
Astaxanthin effectively improves ovarian reserve function in mice. **A** Ovary weight and ovarian index of mice in Control, POF, and POF + AST groups (n = 5). **B** Representative HE staining of murine ovaries. scale bar: 50 μm. **C**-**F** Follicle counts based on serial ovary sections(n = 5). ***p < 0.001compared with the control group, ##p < 0.01, ###p < 0.001 compared with the POF group, one-way ANOVA

To comprehensively assess the therapeutic potential of astaxanthin for ovarian function in POF, we evaluated key morphological and histopathological parameters. The ovarian index was significantly increased in the 200 mg/kg and 300 mg/kg groups compared with the POF group (Fig. 2A). Hematoxylin–eosin (H&E) staining revealed that ovarian architecture in the 100 mg/kg group remained indistinguishable from that of the POF group, with no discernible improvement in follicular integrity or stromal organization (Fig. 2B). In contrast, ovaries from mice treated with 200 or 300 mg/kg astaxanthin exhibited marked structural preservation, including increased cortical follicle density and reduced stromal fibrosis. Quantitative follicle counting demonstrated that significant depletion of primary and antral follicles (Fig. 2 C–D) and a concomitant increase in atretic follicles in the POF group compared with healthy controls (Fig. 2E). Astaxanthin treatment at 100 mg/kg did not reverse these abnormalities. However, both the 200 and 300 mg/kg doses significantly increased antral follicle counts and reduced the proportion of atretic follicles compared with the POF group (Fig. 2F), indicating a dose-dependent restoration of follicular oogenesis and suppression of follicular atresia.

### Astaxanthin reduces ovarian apoptosis and oxidative stress in CTX-induced POF mice

**Fig. 3:**
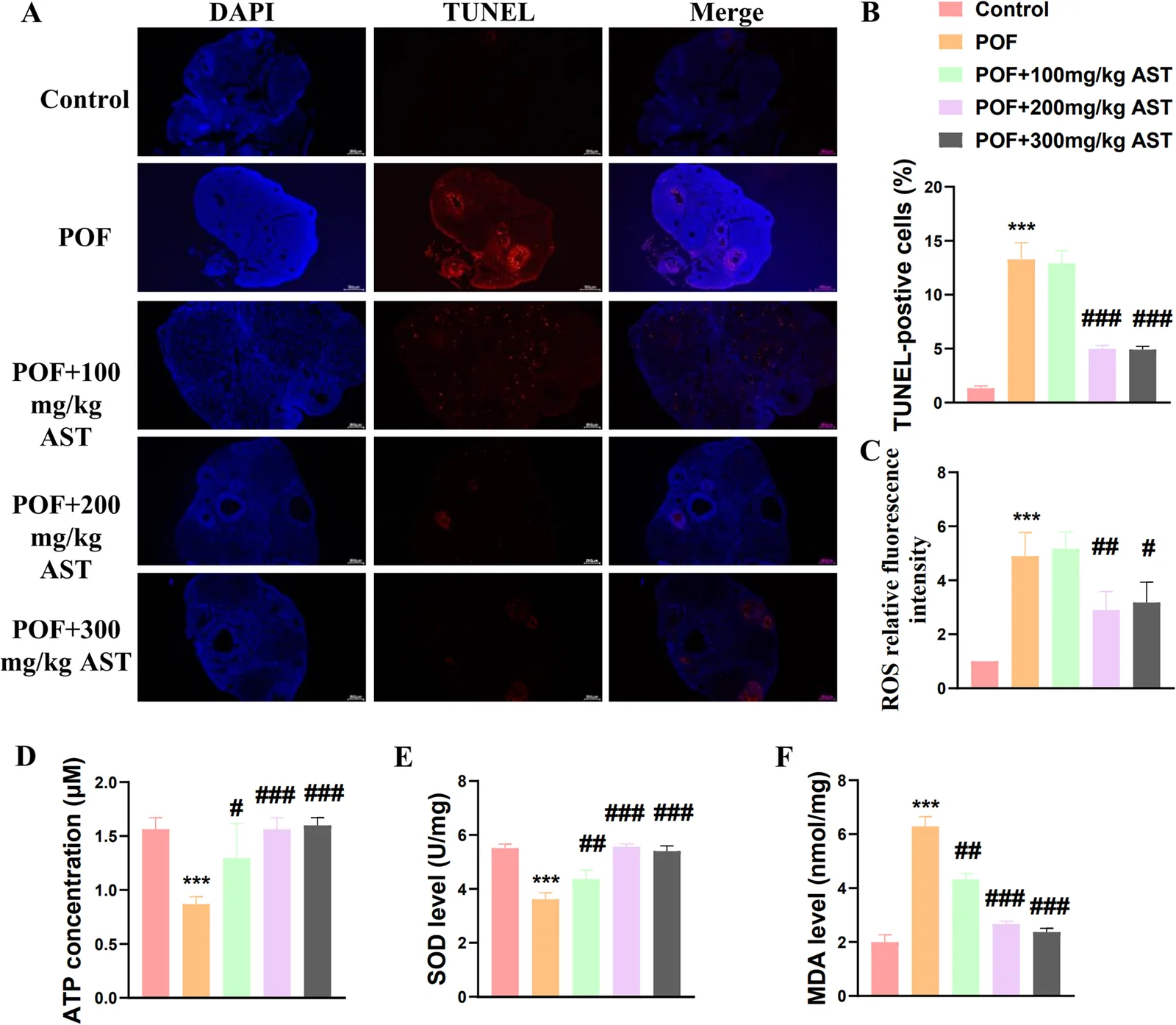
Effect of astaxanthin on CTX induced apoptosis of granulosa cells in ovary. **A** Apoptosis of granulosa cells was analyzed using in situ TUNEL fluorescence staining. Red fluorescence indicates TUNEL-positive (apoptotic) cells. **B** The number of TUNEL-positive granulosa cells and the total granulosa cell count within antral follicles were quantified, and the apoptosis rate of granulosa cells was compared among different groups. **C** quantification of ROS levels measured with a multifunctional microplate reader. **D**-**F** Effect of astaxanthin on oxidative stress. The intracellular ATP activity (**D**), SOD activity (**E**), and MDA levels (**F**) in ovary. The scale bars represent 200 μm. All results are presented as the mean ± standard deviation of at least three independent experiments. Different letters above the columns indicate signiffcant differences (*P* < 0.05). The same letters above the columns indicate non-significant differences(*n* = 5). ***p < 0.001compared with the control group, #*P* < 0.05, ##*p* < 0.01, ###*p* < 0.001 compared with the POF group, one-way ANOVA

Ovarian granulosa cells constitute the somatic cell layer surrounding oocytes within developing follicles and play essential roles in folliculogenesis, steroidogenesis, and oocyte maturation [32, 33]. Excessive granulosa cell apoptosis triggers follicular atresia and compromises ovarian reserve. To assess ovarian functional integrity, we quantified granulosa cell apoptosis in antral follicles using the terminal deoxynucleotidyl transferase dUTP nick-end labeling (TUNEL) assay. Compared with the control group, the number of TUNEL-positive granulosa cells was a significantly increased in the POF group. After astaxanthin treatment, the apoptosis rate in the 100 mg/kg treatment group showed no significant difference compared with the POF group, whereas both the 200 mg/kg and 300 mg/kg groups demonstrated a significant decrease in granulosa cells apoptosis relative to the POF group (Fig. 3A, B).

Consistent with the involvement of oxidative stress in POF pathogenesis, the POF group displayed significantly elevated reactive oxygen species (ROS) levels in ovarian tissue compared with the control group, whereas astaxanthin administration at 200 and 300 mg/kg significantly decreased ROS levels (Fig. 3 C). Furthermore, serum ATP concentrations, which reflect mitochondrial bioenergetic capacity, were significantly decreased in the POF group. After treatment with astaxanthin, ATP levels were increased in all treatment groups, with more pronounced increases in the 200 and 300 mg/kg groups (Fig. 3D).

To further evaluate antioxidant defense status, we measured superoxide dismutase (SOD) activity and malondialdehyde (MDA) concentration in ovarian homogenates. SOD, a key endogenous antioxidant enzyme that catalyzes the dismutation of superoxide radicals (O₂•⁻) into O₂ and H₂O₂, was significantly downregulated in the POF group compared with the control group. All astaxanthin-treated groups (100, 200, and 300 mg/kg) exhibited significantly higher SOD activity than the POF group (Fig. 3E). Conversely, the MDA levels in the POF group significantly increased (Fig. 3 F). After treatment with astaxanthin, the 100 mg/kg, 200 mg/kg and 300 mg/kg treatment groups all significantly reduced MDA levels in the ovarian tissues.

### Astaxanthin modulates apoptosis-related and antioxidant protein expression in ovarian tissue of CTX-induced POF mice

**Fig. 4:**
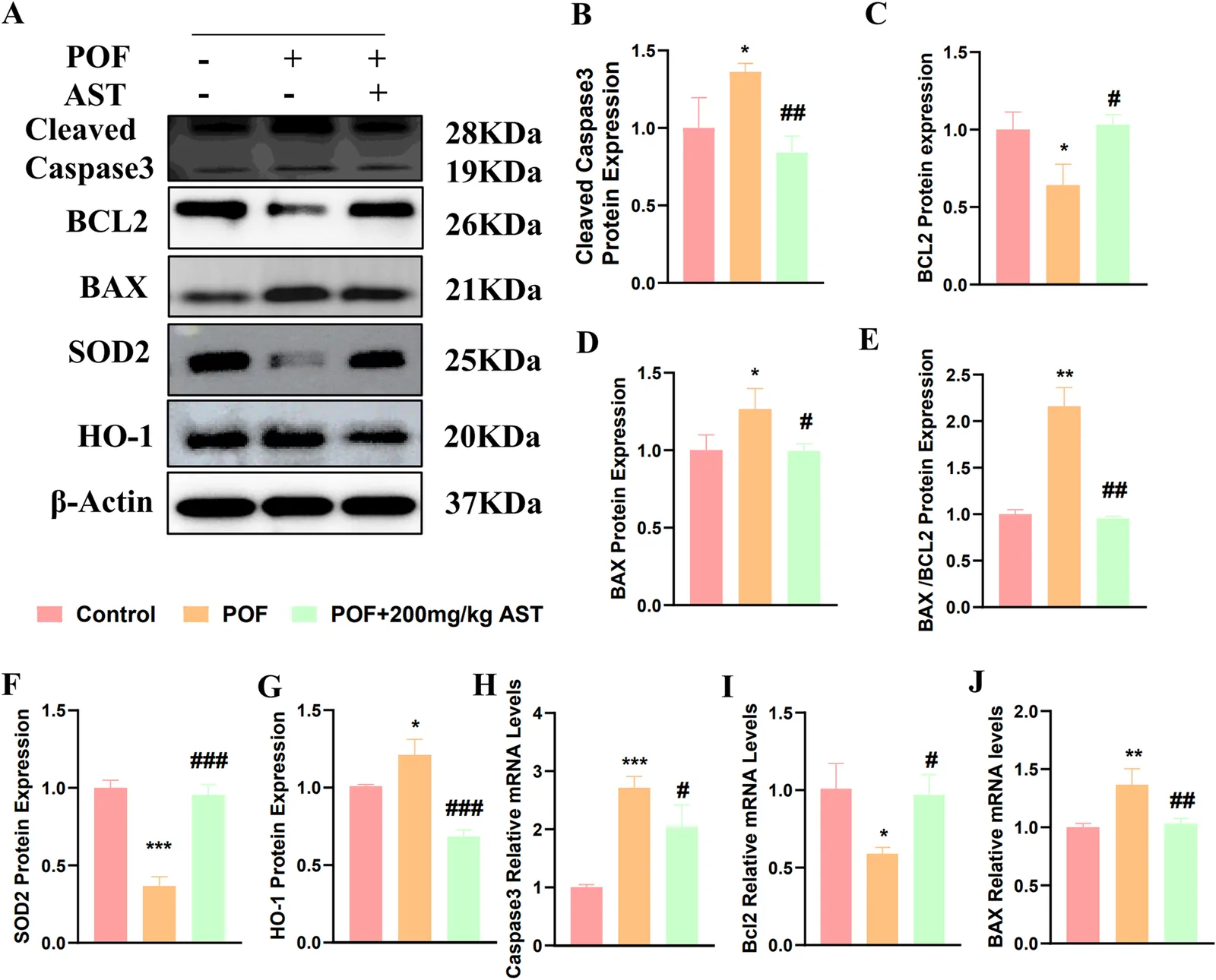
Astaxanthin significantly inhibits apoptosis in POF mice in vivo. **A**-**G** Protein levels of BAX、BCL2, and Cleaved-Caspase3, SOD2, HO-1 were analyzed by Western blot in ovarian tissue; all experiments were repeated at least three times, and the results of representative experiments were shown. **H**-**J**. RT-PCR analyzed the mRNA levels of Caspase3 and BCL2 in ovarian tissues; all experiments were repeated at least three times, and the results of representative experiments were shown. *p < 0.05, **p < 0.01 ***p < 0.001compared with the control group, #*P* < 0.05, ##*p* < 0.01 compared with the POF group, one-way ANOVA

We assessed the expression of key regulators of cell proliferation, apoptosis, and redox homeostasis in ovarian tissues using quantitative real-time PCR (RT-qPCR) and Western blot analysis. Compared with the POF group, the level of cleaved-Caspase 3 and BAX showed significantly downregulated in 200 mg/kg astaxanthin treatment, while BCL2 expression was significantly up-regulated (Fig. 4A-D, H-J). Consequently, the decreased BAX/BCL2 ratio was marked reduced in the 200 mg/kg group (Fig. 4E), indicating a net inhibition of the intrinsic apoptotic pathway. Additionally, the level of HO-1 proteins was reduced, whereas SOD2 protein level was increased in 200 mg/kg group compared with the POF group (Fig. 4F and G), collectively suggests that astaxanthin can effectively ameliorate redox stability and decrease oxidative stress within ovary of mice.

### Astaxanthin restores autophagy in ovarian tissue of CTX-induced POF mice.

**Fig. 5:**
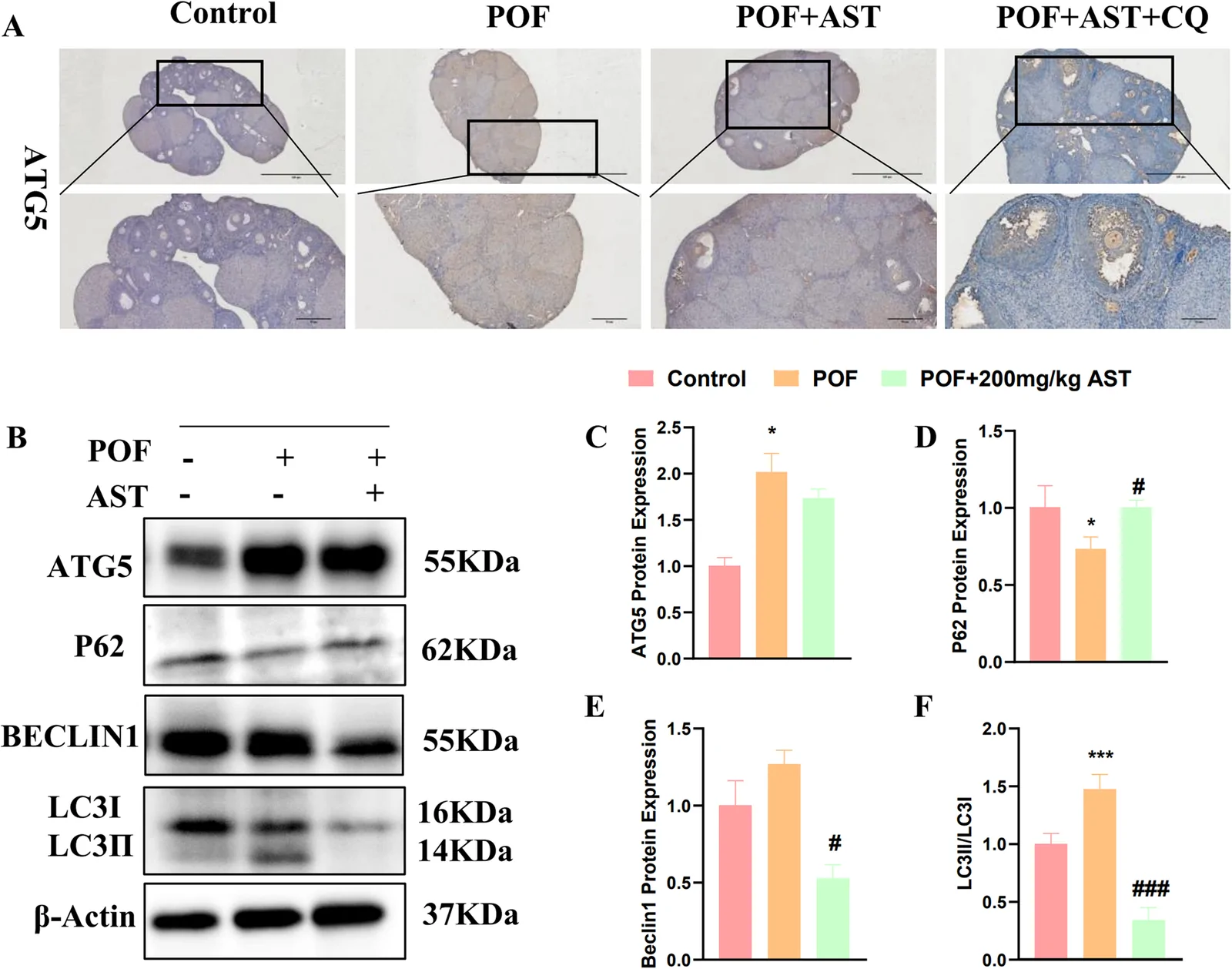
Impact of astaxanthin on autophagy processes in ovary of mice. **A** Immunohistochemical staining of ovaries in Control, POF, and POF + AST groups, scale bar:100 μm, 50 μm. **B**-**F** Protein levels of ATG5, P62, BECLIN1, and LC3 were analyzed by Western blot in ovarian tissue; all experiments were repeated at least three times, and the results of representative experiments were shown. *p < 0.05, ***p < 0.001, ****p < 0.0001compared with the Control group. #*P* < 0.05, ###*p* < 0.001 compared with the POF group, one-way ANOVA

To assesses the modulatory effect of astaxanthin on ovarian autophagy in vivo, immunohistochemical (IHC) staining for autophagy-related protein 5 (ATG5) was performed on ovarian tissue sections from treated mice. IHC staining showed markedly reduced ATG5 expression in the 200 mg/kg/day astaxanthin compared with the POF group (Fig. 5A). Western blot analysis also showed a pronounced decrease in ATG5 protein levels in the ovaries of the 200 mg/kg/day astaxantin-treated mice (Fig. 5B and C). Concurrently, we detected elevated p62/SQSTM1 protein levels, a decreased LC3II/LC3I ratio, and reduced BECLIN-1 expression in the same group (Fig. 5B, D-F). These findings verified that astaxanthin restores autophagic flux homeostasis and effectively ameliorates excessive autophagy in POF mice.

### Network pharmacology forecasts the signaling pathway of Astaxanthin in POF

**Fig. 6:**
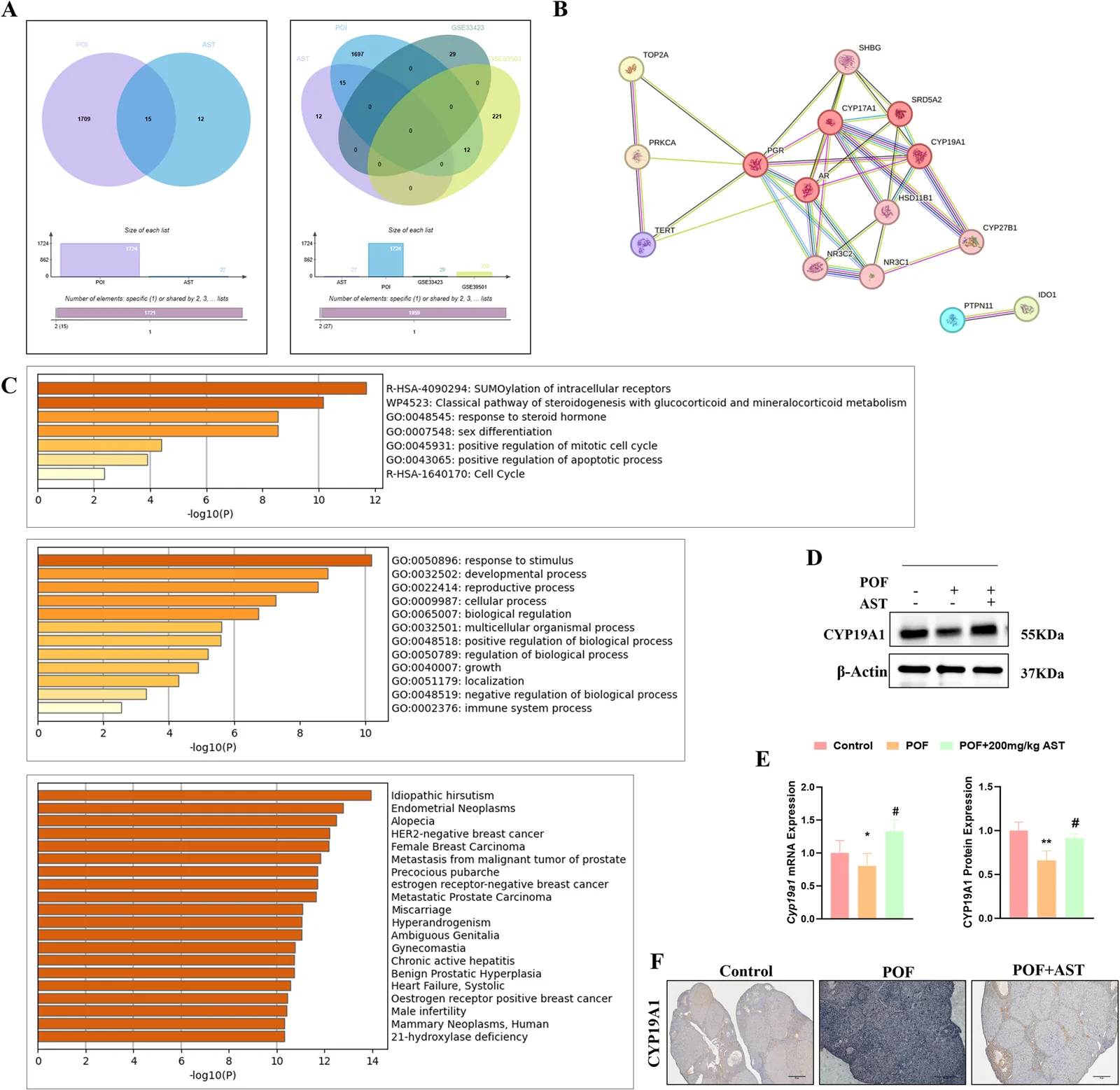
Network pharmacology predicts the signaling pathway of astaxanthin in POF. **A** The 15 targets overlapped 1724 between astaxanthin and POF, regarding as the astaxanthin -regulated POF targets. **B** The protein-protein interaction network based on the 15 overlapped targets. **C** The top 20 biological process (BP), molecular function (MF) and cellular component (CC) in GO enrichment and KEGG enrichment analysis. **D** Protein level of CYP19A1 was analyzed by Western blot in ovarian tissue. **E** RNA level of CYP19A1 was analyzed by q-PCR in ovarian tissue. **F **IHC staining of CYP19A1 in different ovarian tissue. Aall experiments were repeated at least three times, and the results of representative experiments were shown. *p < 0.05, **p < 0.001, compared with the Control group. #P < 0.05 compared with the POF group, one-way ANOVA

Based on the above results, it is shown that Astaxanthin can effectively inhibit apoptosis in CTX-induced POF mice. However, the underlying mechanisms remain unclear. Thus, network pharmacology was performed to predict the potential targets and signaling pathways of astaxanthin in POF. Firstly, 6929 astaxanthin-related targets and 1724 POF-related targets were retrieved from online databases, yielding 15 overlapping targets between astaxanthin and POF (Fig. 6A). These intersecting targets were selected as astaxanthin-regulated POF targets. Secondly, a protein-protein interaction (PPI) interactions of the 15 crossing targets was established by the STRING database (Fig. 6B). These hub targets, including AR, CYP19A1, CYP17A1, SRDSA2, PGR, and others, might be the most beneficial for astaxanthin against POF. Among these hub targets, CYP19A1 ranked among the top hub targets by degree centrality. Subsequently, Enrichment analysis using GO and KEGG pathway was carried out based on the astaxanthin-regulated POF targets. According to GO enrichment analysis, these targets were predominantly enriched in positive regulation of programmed cell cycle, response to steroid hormone, response to oxygen levels, regulation of apoptotic signaling pathway, and others (Fig. 6C). Among these, “response to steroid hormone” is particularly relevant, as CYP19A1 encodes aromatase—the rate-limiting enzyme in estrogen biosynthesis.

To experimentally validate the prediction, we examined CYP19A1 expression in ovarian tissue. RT-qPCR and Western blot analysis revealed that astaxanthin treatment (200 mg/kg) significantly upregulated CYP19A1 mRNA and protein levels compared with the POF group (Fig. 6D, E). IHC staining further verified this incraese (Fig. 6F). Collectively, these above results indicate that astaxanthin improves ovarian reserve function by modulating CYP19A1-estrogen axis.

## Discussion

Premature ovarian failure (POF) has emerged as a significant global concern, adversely affecting family well-being and social harmony [34, 35]. Cyclophosphamide (CTX) is a commonly used chemotherapeutic drug for breast cancer, featuring high cost-effectiveness and strong inhibitory effects. However, its significant reproductive toxicity adversely affects the reproductive health of patients undergoing chemotherapy. More than half of breast cancer patients experience adverse reactions after CTX treatment, such as menstrual disturbances or even amenorrhea, which are clinical and pathological characteristics of POF [6, 7]. What’s more, effective strategies for preventing and treating CTX-induced POF remain to be developed.

Astaxanthin, a natural antioxidant, possesses potent cytoprotective, anti-inflammatory, anti-tumor, and anti-aging properties [8, 36]. Previous studies have shown that astaxanthin reduces apoptosis and alleviate oxidative stress in mice using a model of polycystic ovarian disease [37, 38]. In the present study, we investigated the regulatory effects of astaxanthin on CTX-induced ovarian reserve dysfunction in KM mice. We found that astaxanthin administration by gavage significantly improved estrous cycle disorders and restored serum levels of anti-Müllerian hormone (AMH) and estradiol (E2), suggesting that astaxanthin improved reproductive endocrine function. Furthermore, ovarian SOD activity was significantly decreased, whereas MDA levels were significantly increased, indicating oxidative stress damage. Astaxanthin treatment significantly reversed these changes, demonstrating its ability to alleviate oxidative stress injury in the mouse ovaries.

In addition, our results showed that apoptosis of ovarian granulosa cells was increased in the POF group. Astaxanthin treatment significantly reduced ovarian apoptosis. Histological analysis further revealed a significant decrease in atretic follicles (ATF) and a significant increase in primary (PF) and antral follicles (AF). It has been reported that apoptosis plays a dominant role in follicular atresia at the terminal stage of development [39]. Based on these findings, we speculate that CTX-induced ovarian damage in mice is primarily mediated by oxidative stress-induced apoptosis, resulting in increased follicular atresia. Astaxanthin exerts a positive effect on the antioxidant stress of ovaries, thereby improving ovarian function.

Autophagy is a dynamic process involving autophagosome formation, fusion with lysosomes to form auto-phagolysosomes. Dysregulation at any of these stages can lead to cellular damage [19, 38]. Our studies showed that astaxanthin administration dramatically decreased LC3II/LC3I ratio and increased P62 levels, indicating that it mitigated ovarian tissue damage by reducing autophagic accumulation and restoring autophagic flux.

Network pharmacology and KEGG enrichment analysis identified“steroid hormone”, “Cell Cycle”, and “Reproductive process” as the core pathways involved in astaxanthin-mediated protection against CTX-induced POF. Notably, CYP19A1, the rate-limiting enzyme in estrogen biosynthesis, emerged as a central node within the ovarian steroidogenesis pathway and was predicted as a direct target of astaxanthin. Consistent with this prediction, astaxanthin treatment significantly restored serum estradiol (E2) levels and upregulated ovarian CYP19A1 expression in POF mice. However, a growing body of evidence supports a bidirectional regulatory relationship between CYP19A1/E2 and autophagy. On one hand, inhibition of autophagy in granulosa cells has been shown to downregulate CYP19A1/aromatase expression and reduce E2 synthesis through impaired degradation of the transcription factor WT1 [25]. On the other hand, E2, via activation of estrogen receptors (ERα/ERβ), can induce autophagy through the AKT/mTOR pathway, forming a positive feedback loop that maintains granulosa cell homeostasis [23, 24]. The concurrent enrichment of “Estrogen signaling pathway” and "Apoptosis" in our KEGG analysis further supports the existence of this regulatory network.

**Fig. 7:**
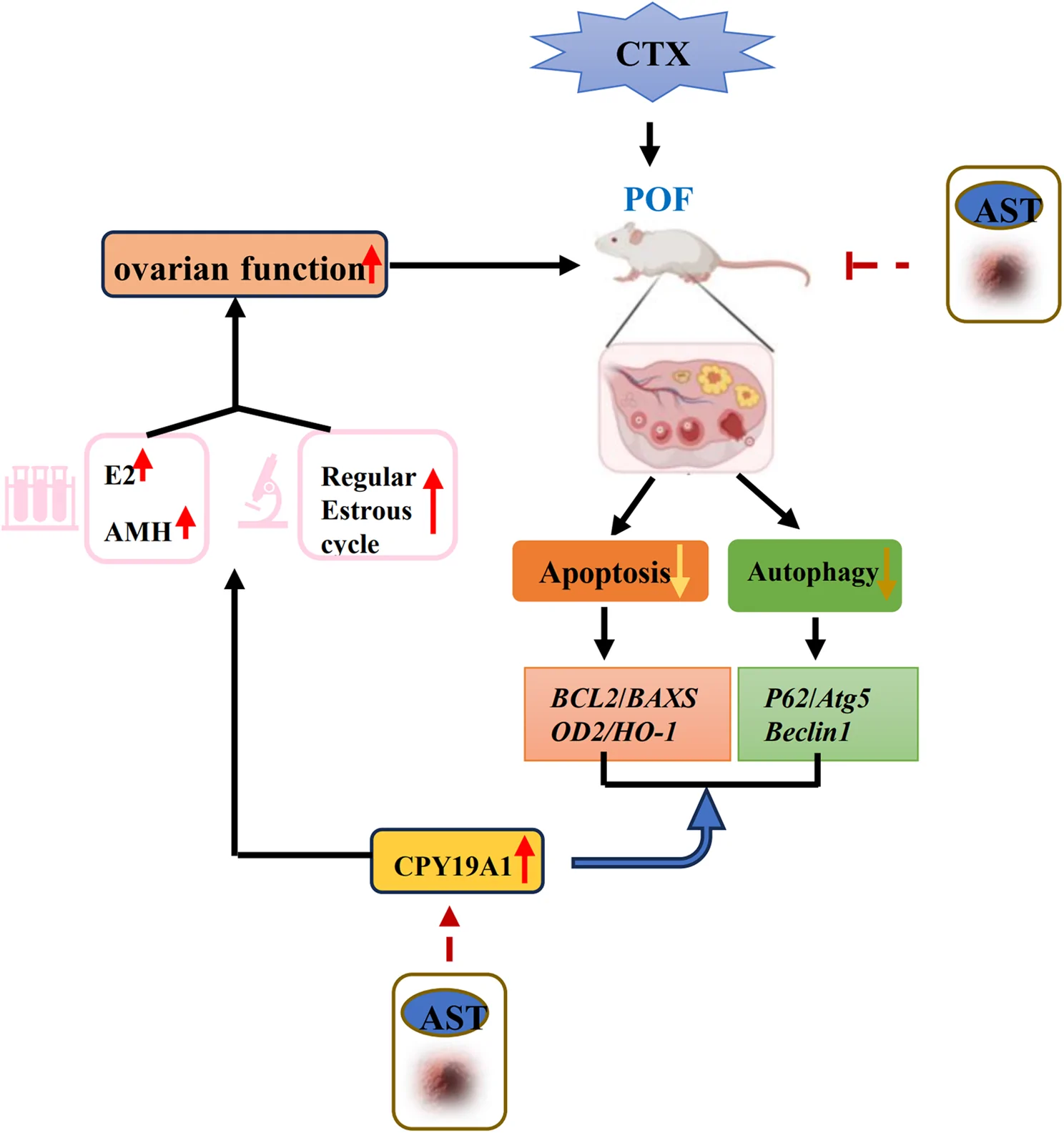
Schematic diagram of the proposed model by which astaxanthin attenuates CTX-induced premature ovarian failure by modulating granulosa cell apoptosis and autophagy

Based on these findings, we propose a model in which astaxanthin directly targets CYP19A1, restoring E_2_ synthesis and activating ER-mediated signaling, which in turn normalizes autophagic-lysosome function and inhibits granulosa cell apoptosis, ultimately preserving ovarian reserve. The following studies should include in vitro validation using primary granulosa cells or cell lines (e.g., KGN or COV434) to investigate the mechanism by which astaxanthin, through the CYP19A1, affects the apoptosis and autophagy of granular cells. In conclusion, this study confirmed that astaxanthin partially alleviated the oxidative stress damage to ovarian function caused by CTX, and might play a protective role in ovarian function through the CYP19A1, providing new insights and experimental basis for the treatment of POF (Fig. 7). However, the specific regulatory mechanism requires needs further elucidation. Future studies will focus on elucdating the molecular mechanism by which CYP19A1 mediates ovarian protection.

## Supplementary Information

Supplementary Material 1.

## Data Availability

No new data were created or analysed in this study. Data sharing is not applicable to this article.
